# Effect of ceritinib on the pharmacokinetics of coadministered CYP3A and 2C9 substrates: a phase I, multicenter, drug–drug interaction study in patients with *ALK* + advanced tumors

**DOI:** 10.1007/s00280-020-04180-3

**Published:** 2021-01-04

**Authors:** Felipe K. Hurtado, Filippo de Braud, Javier De Castro Carpeño, Maria Jose de Miguel Luken, Ding Wang, Jeffrey Scott, Yvonne Y. Lau, Tracey McCulloch, Morten Mau-Sorensen

**Affiliations:** 1 Novartis Pharmaceuticals Corporation, East Hanover, NJ USA; 2 Fondazione IRCCS-Istituto Nazionale Dei Tumori, Milano, Italy; 3 Medical Oncology Department, Hospital Universitario La Paz, IdiPAZ, Madrid, Spain; 4 CIOCC-Grupo Hospitalario de Madrid, Hosp. de Sanchinarro, Madrid, Spain; 5 Henry Ford Cancer Institute, Detroit, MI USA; 6 Department of Oncology, Rigshospitalet, Copenhagen, Denmark

**Keywords:** ALK inhibitor, Ceritinib, Drug–drug interaction, Pharmacokinetics, CYP3A, CYP2C9, Midazolam, Warfarin

## Abstract

**Purpose:**

Ceritinib is an ALK receptor tyrosine kinase inhibitor approved as first- and second-line treatment in adult patients with *ALK* + metastatic non-small cell lung cancer (NSCLC). The study investigated the drug–drug interaction (DDI) potential of ceritinib when coadministered with midazolam and warfarin as probe substrates for CYP3A and CYP2C9 activity, respectively.

**Methods:**

This was a phase I, multicenter, open-label, single sequence, crossover DDI study in 33 adult patients with *ALK* + NSCLC or other advanced tumors. A single dose of a cocktail consisting of midazolam and warfarin was administered with and without concomitant administration of ceritinib. The primary objective was to evaluate the pharmacokinetics of midazolam and warfarin. Secondary objectives included pharmacokinetics, safety, tolerability, overall response rate (ORR), and duration of response (DOR) of ceritinib 750 mg once daily.

**Results:**

Ceritinib inhibited CYP3A-mediated metabolism of midazolam, resulting in a markedly increased AUC (geometric mean ratio [90% confidence interval]) by 5.4-fold (4.6, 6.3). Ceritinib also led to an increase in the AUC of S-warfarin by 54% (36%, 75%). The pharmacokinetics and safety profile of ceritinib in this study are consistent with previous reports and no new safety signals were reported. Among the 19 patients with NSCLC, efficacy (ORR: 42.1% and DCR: 63.2%) was similar to that reported previously in studies of pretreated patients with *ALK* + NSCLC.

**Conclusion:**

Ceritinib is a strong CYP3A inhibitor and a weak CYP2C9 inhibitor. These findings should be reflected as actionable clinical recommendations in the prescribing information for ceritinib with regards to concomitant medications whose pharmacokinetics may be altered by ceritinib.

**Electronic supplementary material:**

The online version of this article (10.1007/s00280-020-04180-3) contains supplementary material, which is available to authorized users.

## Introduction

Anaplastic lymphoma kinase (*ALK*)-rearrangements are a key oncogenic driver, implicated in the pathogenesis of several human cancers [1]. In non-small-cell lung cancer (NSCLC), *ALK*-rearrangements have been reported in approximately 3–7% of patients [2, 3].

Ceritinib (LDK378, Zykadia®; Novartis Pharmaceuticals Corporation) is an orally available ALK inhibitor, approximately 20-fold more potent than crizotinib [4]. Ceritinib is approved at a dose of 450 mg taken orally once daily (QD) with food for the treatment of patients with metastatic *ALK*-positive NSCLC [5]. In randomized, global phase III trials, ceritinib demonstrated a statistically significant and clinically meaningful improvement in progression-free survival vs. chemotherapy in patients with advanced *ALK*-positive NSCLC who were treatment-naïve (ASCEND-4 study) [6] or previously treated with crizotinib and one or two prior chemotherapy regimens (ASCEND-5 study) [7].

Ceritinib is primarily cleared by hepatic oxidative metabolism via CYP3A (> 90%) as demonstrated in in vitro human liver microsome (HLM) studies. The metabolic drug–drug interaction (DDI) potential of ceritinib as an inhibitor of CYP metabolizing enzymes was evaluated in vitro by incubation with pooled HLMs. Ceritinib competitively inhibited the metabolism of midazolam (CYP3A substrate) and diclofenac (CYP2C9 substrate). Furthermore, ceritinib is a reversible and time-dependent inhibitor of CYP3A (maximum rate of inactivation [k_inact_]: 0.0642 min-1; inhibitor concentration associated with 50% maximal inactivation rate [Ki]: 1.47 μM), but shows no apparent time-dependent inhibition of CYP1A2, 2C9, or 2D6 at ceritinib concentrations up to 50 µM. Ceritinib is unlikely to inhibit CYP1A2, 2B6, 2C8, 2C19, or 2D6 at clinically relevant concentrations [8].

Inhibition of CYP3A- and CYP2C9-mediated metabolism by ceritinib can lead to a decreased rate of elimination of concomitantly administered drugs that are primarily metabolized by these isoforms, resulting in an increased systemic exposure and potential toxicity, especially for those drugs that have a narrow therapeutic index (NTI). To provide actionable labeling recommendations to healthcare practitioners for safe and effective use of co-administered CYP3A and 2C9 substrates with ceritinib, the extent of CYP inhibition by ceritinib needs to be further characterized considering the potential of clinically relevant drug interactions. 

Evaluation of a candidate drug’s potential for in vivo induction or inhibition of CYPs and transporters by the administration of a drug cocktail allows simultaneous assessment of multiple drug–substrate interactions under controlled conditions [9–11]. Turpault et al. [10] reported the absence of pharmacokinetic interaction between warfarin (CYP2C9 substrate) and midazolam (CYP3A substrate) when administered concurrently in a clinical trial conducted in healthy male subjects. Since CYP3A subfamily enzymes are predominantly expressed in the liver and intestine in humans, co-administration of oral ceritinib may alter both intestinal absorption and fraction escaping hepatic metabolism (i.e., bioavailability) of an orally administered CYP3A substrate. As a result, oral rather than intravenous midazolam was the preferred route of administration in this study.

This study was designed to evaluate the potential inhibitory effects of ceritinib on the CYP3A- and CYP2C9-mediated metabolism of the probe drugs midazolam and warfarin, respectively, when administered simultaneously as a cocktail in patients with *ALK*-positive tumors. The results of this clinical trial provided evidence on how to dose CYP3A and 2C9 substrates when administered concomitantly with ceritinib.

## Materials and methods

### Study population

Adult patients (≥ 18 years) with confirmed stage IIIB or IV *ALK*-positive NSCLC or an advanced tumor other than NSCLC that carried an *ALK* genetic alteration were eligible to participate in the study. *ALK* positivity was determined locally using an approved test. Other inclusion criteria included a World Health Organization performance status (WHO PS) of 0–2, adequate organ function as assessed by laboratory test results, and asymptomatic or neurologically stable central nervous system metastases. Eligible patients were either treatment-naïve or were previously treated with ≥ 1 systemic anticancer therapy (including chemotherapy, other *ALK* inhibitors, or immunotherapy).

Usage of concomitant drugs that could confound the study results or expose patients to risk for drug-related toxicity had to be discontinued ≥ 4 weeks prior to study start and for the duration of the study, including but not limited to strong inhibitors or inducers of CYP3A and/or CYP2C9, medications with a low therapeutic index that are primarily metabolized by CYP3A and/or CYP2C9, and medications with a known risk of causing QT prolongation and/or Torsades de pointes. Patients who consumed alcohol (regular daily intake ≥ 1 drink/day) or grapefruit/grapefruit juice ≤ 3 days prior to pharmacokinetic blood sample collection were also excluded.

### Study design

**Fig. 1:**
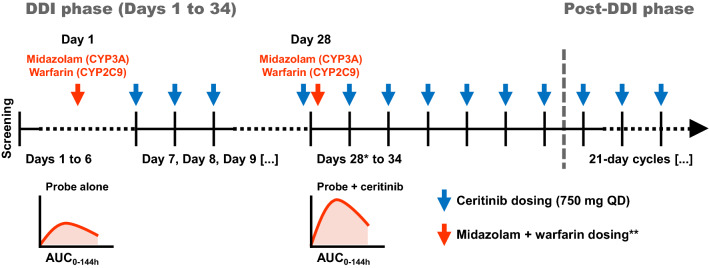
Study design. *Ceritinib steady-state was reached by Day 28 following 3 weeks of daily dosing (see Figure S2). **Midazolam and warfarin administered as a single oral dose of 2.5 mg and 10 mg, respectively. *AUC* area under the plasma concentration–time curve, *DDI* drug–drug interaction, *QD* once daily

This was a multicenter, open-label, single sequence, crossover phase I study (ClinicalTrials.gov identifier: NCT02422589) and was divided into two phases, DDI phase and post-DDI treatment phase (Fig. 1). Patients were recruited from 10 clinical centers located across 4 countries, namely Spain (*n* = 3), United States (*n* = 3), Italy (*n* = 3), and Denmark (*n* = 1). On Day 1 of the DDI phase, patients were administered a single dose of the probe drug cocktail comprising warfarin (10 mg) and midazolam (2.5 mg) and a full pharmacokinetic profile for each probe drug was collected over 144 h post-dose. Ceritinib (750 mg orally QD under fasted conditions) was administered continuously for 3 weeks starting from Day 7 following the collection of last pharmacokinetic blood sample to reach steady-state and hence max out the inhibitory effect towards CYP-metabolizing enzymes. On Day 28, patients received a dose of 750 mg ceritinib and a concomitant single dose of the midazolam + warfarin cocktail, followed by collection of a full pharmacokinetic profile. The individual pharmacokinetic profiles of probe drugs administered on Day 28 were compared to those acquired on Day 1 to assess the extent of CYP3A and CYP2C9 inhibition by ceritinib.

Upon completion of the DDI phase on Day 34, patients entered the post-DDI phase and continued to be treated with ceritinib at a dose of 750 mg QD on continuous 21-day cycles until unacceptable toxicity, disease progression, withdrawal of consent, or at the discretion of the investigator. Patients were allowed to receive treatment with ceritinib following disease progression if, in the opinion of the investigator, continued treatment provided clinical benefit. Ceritinib dose reductions (150 mg/day per dose reduction step, up to the minimum allowed dose of 300 mg/day) were permitted for patients who did not tolerate the 750 mg/day starting dose.

The primary objective of the study was to evaluate the pharmacokinetics of midazolam and warfarin, with and without concomitant administration of ceritinib. Secondary objectives were to assess the pharmacokinetics, safety, tolerability, and antitumor activity (by means of overall response rate [ORR] and duration of response [DOR]) of ceritinib in patients with advanced *ALK*-positive tumors.

The ceritinib dose chosen for this study was 750 mg QD fasted, which was the dose used in the pivotal clinical study that served as basis for the regulatory approval of ceritinib in *ALK* + NSCLC, as well as the original dose approved in most countries. On the basis of pharmacokinetics, safety, and efficacy results from a post-marketing dose optimization study conducted in *ALK* + NSCLC patients [15, 16] (ASCEND-8 study), the recommended dose of ceritinib was changed to 450 mg QD with food. The data acquired in ASCEND-8 study demonstrated equivalent steady-state exposure of ceritinib between 450 mg QD with food vs. 750 mg QD fasted, in addition to the 450 mg with food dose having the more favorable gastrointestinal safety profile with similar efficacy to 750 mg fasted. Therefore, the results generated in the present DDI study (which used ceritinib 750 mg QD fasted) pertaining to changes in exposure of co-administered CYP3A and 2C9 substrates are directly applicable to the newly approved dose of ceritinib 450 mg QD with food.

The study was designed and conducted in accordance with the ethical principles of the Declaration of Helsinki and the guidelines for Good Clinical Practice, with applicable local regulations. The study protocol and all amendments were reviewed by an independent ethics committee or institutional review board for each center. All patients provided written informed consent before screening.

### Assessments

To assess the pharmacokinetics of probe substrates, blood samples were obtained on Days 1 and 28 at pre-dose and at 0.25, 0.5, 1, 2, 3, 4, 6, 8, 10, 24, 48, 72, 96, 120, and 144 h post-dose. Additional blood samples were obtained at pre-dose on Days 28, 29, 30, 31, 32, 33, and 34 to assess steady-state pharmacokinetics of ceritinib. Plasma concentrations of ceritinib were measured by a validated liquid chromatography-tandem mass spectrometry (LC–MS/MS) assay with a lower limit of quantification (LLOQ) of 1.00 ng/mL using 100 µL of human plasma [12]. Plasma concentrations of midazolam, 1′-hydroxymidazolam (LLOQ of 0.100 ng/mL), S-warfarin, R-warfarin, and 7-hydroxy-S-warfarin (LLOQ of 2.00 ng/mL) were also determined using validated LC–MS/MS assays. Pharmacokinetic parameters of midazolam, warfarin, and their metabolites were determined by non-compartmental analysis using Phoenix WinNonlin version 6.4 (Certara Inc., Princeton, NJ, USA). A blood sample for CYP2C9 genotyping was collected on Day 1 of the DDI phase from all participating patients. Patients were categorized as CYP2C9 poor, intermediate, or extensive metabolizers based on the genotype testing results. This assessment was of exploratory nature and was not used as inclusion/exclusion criteria in the study.

Adverse events (AEs) were coded using the Medical Dictionary for Regulatory Activities version 20.1 and were graded using Common Terminology Criteria for Adverse Events version 4.03. All patients were followed up for AEs and serious AEs (SAEs) for at least 30 days following the last dose of study treatment at the end of treatment phase. Tumor evaluation was performed at screening visit within 28 days prior to study treatment (Day 1 of DDI phase) using contrast enhanced computed tomography (or magnetic resonance imaging). Assessments of tumor response were performed every two cycles (i.e., every 6 weeks) through Cycle 9. ORR and DOR by investigator assessment was reported per Response Evaluation Criteria in Solid Tumors (RECIST) 1.1.

### Statistical analysis

A sample size of 18 patients with evaluable pharmacokinetic data was determined to provide reasonable precision in the estimation of geometric mean ratio (GMR) assuming the intra-subject CV of warfarin (20%) and midazolam (18.5%) pharmacokinetics. Due to the strict evaluability criteria and the requirement to conduct the study with repeat daily doses of ceritinib, the total number of patients enrolled was targeted to be approximately 35 in order to obtain at least 18 evaluable patients.

The pharmacokinetic analysis set for midazolam and warfarin consisted of all patients who had an evaluable pharmacokinetic profile from both periods (CYP substrate alone and CYP substrate + ceritinib). The criteria used to consider an individual pharmacokinetic profile to be non-evaluable included major deviations in the originally assigned dosing schedule of ceritinib, vomiting within 4 h after dosing, among other pre-defined criteria included in the study statistical analysis plan.

A formal statistical analysis was carried out to compare the single-dose pharmacokinetics of the probe substrates before and after ceritinib administration. A linear mixed-effects model was fitted to the log-transformed primary PK parameters (C_max_, AUC_0-144 h_, and AUC_0-inf_) and included a fixed effect for treatment and a random effect for subject. For the statistical analysis, midazolam/warfarin + ceritinib was the test treatment and midazolam/warfarin alone the reference. The model-based, between-treatment mean differences (probe substrate + ceritinib–probe substrate alone) and corresponding two-sided 90% confidence intervals (CIs) were calculated on the log-scale. The between-treatment differences and 90% CIs were then back-transformed to the original scale to obtain the geometric mean ratios (probe substrate + ceritinib/probe substrate alone) and corresponding 90% CIs.

## Results

### Patient disposition

A total of 33 patients were enrolled in the study; of these, 18 (54.5%) completed the DDI phase. Of the 15 patients who discontinued the DDI phase, 12 patients permanently discontinued from the study, and 3 remained on ceritinib treatment. The majority of patients discontinued from the DDI phase due to AEs or progressive disease (Table S1). All 33 patients received ≥ 1 dose of any study medication and were included in the efficacy and safety analysis sets. Twenty patients (60.6%) were included in the pharmacokinetic analysis set.

### Patient demographics and disease characteristics

The median age of the patients was 57 years (range 22–78), and most were female (63.6%), Caucasian (97.0%), and had a WHO PS of 0 or 1 (94.0%). Baseline patient demographics and disease characteristics for all patients are shown in Table 1. The disease characteristics of patients in this study were representative of the population of patients with *ALK*-positive tumors. The majority of the patients had NSCLC (57.6%), the target indication of ceritinib, and the most common histology/cytology was adenocarcinoma (63.6%). All patients entered the study with stage IV or IVB disease.

**Table 1:** Patient demographics and disease characteristics at baseline

Characteristic	All patients *N* = 33 *n* (%)
Median age (range), years	57.0 (22–78)
Sex, *n* (%)	
Female	21 (63.6)
Male	12 (36.4)
Race, *n* (%)	
Caucasian	32 (97.0)
Unknown	1 (3.0)
WHO performance status, *n* (%)	
0	16 (48.5)
1	15 (45.5)
2	2 (6.1)
Smoking history, *n* (%)	
Current smoker	2 (6.1)
Former smoker	14 (42.4)
Never smoked	17 (51.5)
Primary site of cancer, *n* (%)	
Lung	19 (57.6)
Other	5 (15.2)
Bone, other	2 (6.1)
Skin	2 (6.1)
Soft tissue	2 (6.1)
Abdominal mass	1 (3.0)
Kidney	1 (3.0)
Pelvic	1 (3.0)
Histology/cytology, *n* (%)	
Adenocarcinoma	21 (63.6)
Other	12 (36.4)
Stage at study entry, *n* (%)	
IV	32 (97.0)
IVB	1 (3.0)
Median time from primary diagnosis to enrolment (range), months	22.41 (1.1–185.0)
Number of prior regimens, *n* (%)	
0	3 (9.1)
1	6 (18.2)
2	9 (27.3)
≥ 3	15 (45.5)
Prior anti-cancer medications, *n* (%)	
Chemotherapy	24 (72.7)
ALK inhibitors	
Ceritinib	1 (3.0)
Crizotinib	12 (36.4)
Checkpoint inhibitors	
Ipilimumab	2 (6.1)
Nivolumab	6 (18.2)
Pembrolizumab	1 (3.0)

*ALK* anaplastic lymphoma kinase, *WHO* World Health Organization

### Effect of 750 mg QD ceritinib on the pharmacokinetics of midazolam (CYP3A probe substrate)

**Fig. 2:**
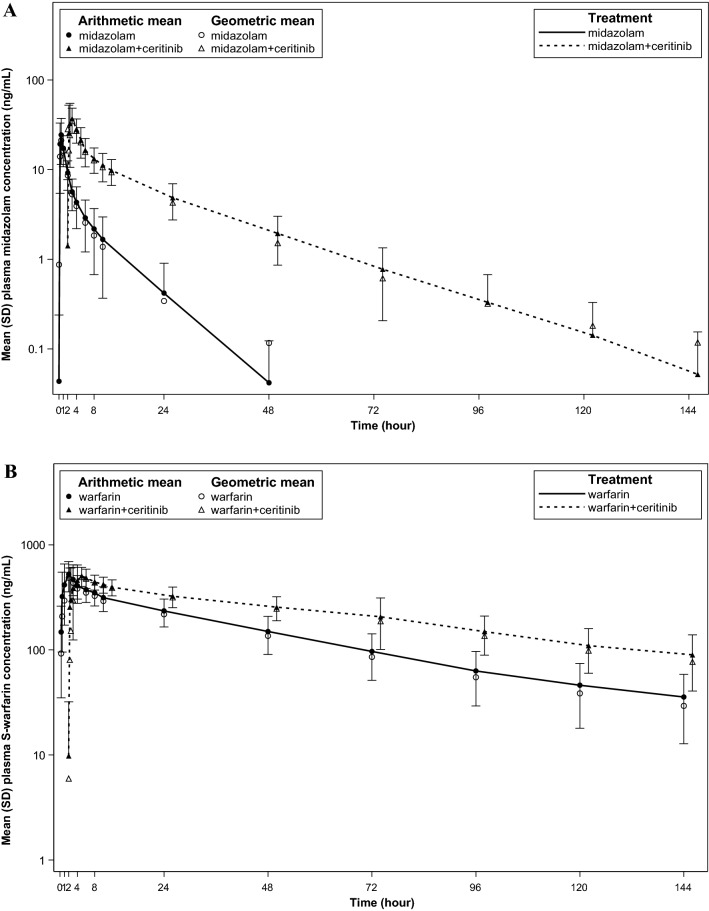
Geometric mean and arithmetic mean (standard deviation) concentration–time profiles depicting the effect of ceritinib on the pharmacokinetics of co-administered CYP3A and CYP2C9 substrates. A Midazolam with and without ceritinib. B S-warfarin with and without ceritinib. *SD* standard deviation

Midazolam exposure increased markedly when co-administered with ceritinib, reflecting an inhibitory effect towards CYP3A activity (Fig. 2). The estimated GMR (90% CI) (midazolam + ceritinib vs. midazolam alone) of midazolam AUC_0-inf_ and *C*_max_ were 5.42 (4.64, 6.34) and 1.82 (1.54, 2.16), respectively (Table S2). The median time to peak plasma concentration (*T*_max_) of midazolam remained unaffected by ceritinib and was reached at approximately 1 h after dosing. Given an increase in midazolam AUC of ≥ 5-fold, these results indicate that ceritinib is a strong inhibitor of CYP3A in the target patient population.

A notable decrease by approximately 80% in the apparent clearance (CL/F) of midazolam (from 34.5 L/h [midazolam alone] to 6.36 L/h [midazolam + ceritinib]) as well as a prolongation of its elimination half-life (*T*_1/2_) from 6 to 15 h confirms the inhibitory effect of ceritinib towards CYP3A. The geometric mean metabolite-to-parent AUC ratio for 1′-hydroxymidazolam (metabolite formed by midazolam 1′-hydroxylation, CYP3A-mediated) decreased from 19.3% (midazolam alone) to 2.9% (midazolam + ceritinib), evidencing a notable reduction in CYP3A-mediated metabolism of midazolam in the presence of ceritinib (Table 2).

**Table 2:** Summary of pharmacokinetic parameters for midazolam, warfarin, and their major metabolites, with and without concurrent administration of ceritinib

CYP probe substrate		*N*	*C*_max_ (ng/mL)	AUC_0–144 h_ (h*ng/mL)	*T*_max_ (h)	CL/F (L/h)	*T*_1/2_ (h)	Metabolite-to-parent ratio (%)^a^
*CYP3A*								
Midazolam		20	23.0 (56.2)	70.2 (46.7)	0.500 [0.217; 1.00]	34.5 (45.8)	6.05 (47.8)	
Midazolam + ceritinib		20	42.0 (39.3)	387 (37.0)	0.750 [0.250; 2.53]	6.36 (35.9)	15.4 (37.4)	
1′-hydroxymidazolam		20	5.41 (74.1)	14.7 (74.4)	0.500 [0.217; 2.00]			19.3 (66.3)
1′-hydroxymidazolam + ceritinib		20	1.90 (73.8)	12.1 (87.0)	0.583 [0.250; 2.53]			2.89 (66.7)
*CYP2C9*								
S-warfarin		20	569 (34.9)	18,200 (46.6)	1.47 [0.483; 3.92]	0.471 (28.1)	42.9 (30.9)	
S-warfarin + ceritinib		20	599 (27.5)	28,400 (36.0)	2.75 [0.250; 6.40]	0.346 (22.8)	55.4 (44.5)	
R-warfarin		20	560 (34.2)	30,600 (39.0)	1.98 [0.483; 21.7]	0.277 (15.6)	62.2 (24.6)	
R-warfarin + ceritinib		20	616 (23.6)	38,500 (34.9)	2.83 [0.250; 71.2]	0.267 (37.6)	77.1 (30.5)	
7-Hydroxy-S-warfarin		20	39.7 (60.3)	2910 (61.8)	24.2 [2.97; 49.9]			14.3 (66.6)
7-Hydroxy-S-warfarin + ceritinib		20	34.9 (61.6)	3320 (64.1)	47.2 [8.50; 73.7]			11.7 (58.2)

Parameters reported as geometric mean (CV%); *T*_max_ reported as median [min; max]

CV% geometric mean = sqrt[exp(variance for log-transformed data) – 1] × 100

*C*_max_: maximum plasma concentration observed; AUC_0–144 h_: area under the plasma concentration–time curve *t* = 0–144 h; *T*_max_: time to reach *C*_max_; *CL/F*: apparent clearance; *T*_1/2_: elimination half-life; *N*: number of subjects included in the analysis

^a^Metabolite-to-parent ratio = [(metabolite AUC_0–144 h_/metabolite molecular weight)/(parent AUC_0–144 h_ / parent molecular weight)] × 100

### Effect of 750 mg QD ceritinib on the pharmacokinetics of warfarin (CYP2C9 probe substrate)

Warfarin is a racemic mixture of S- and R-enantiomers, the former exhibiting 5–6 times greater anticoagulant potency. Hence, pharmacokinetic changes of S-warfarin served as the basis to inform dosing recommendations pertaining co-administration of ceritinib with CYP2C9 substrates, as the pharmacological effect of warfarin resides mainly in S-warfarin. Overall, S-warfarin plasma concentrations were increased in the presence of ceritinib, although the magnitude of increase was not as high as that observed for midazolam (Fig. 2).

The estimated GMR (90% CI) (warfarin + ceritinib vs. warfarin alone) of S-warfarin AUC_0-inf_ and *C*_max_ were 1.54 (1.36, 1.75) and 1.05 (0.912, 1.21), respectively (Table S2). These results indicate that ceritinib is a weak inhibitor of CYP2C9. Although the extent of exposure increase of S-warfarin was < 2-fold, an increase of AUC by 54% is considered clinically relevant, considering warfarin is a widely prescribed anticoagulant for chronic use to treat and/or prevent thromboembolic events in patients and minimal concentration changes in the blood may lead to serious safety concerns. There was no apparent change in the onset of warfarin absorption. S-warfarin CL/F was reduced by approximately 30% and *T*_1/2_ prolonged from 43 to 55 h when co-administered with ceritinib. The geometric mean metabolite-to-parent AUC ratio for 7-hydroxy-S-warfarin (a marker for S-warfarin 7-hydroxylation, CYP2C9-mediated) decreased from 14.3% (warfarin alone) to 11.7% (warfarin + ceritinib), indicating a modest reduction in CYP2C9-mediated metabolism of warfarin in the presence of ceritinib. Ceritinib did not affect the pharmacokinetics of R-warfarin to a clinically significant extent (Table 2, Figure S1).

Patient disposition by CYP2C9 metabolizer subtype is shown in Table 3. The majority of the patients were categorized as CYP2C9 extensive metabolizers (51.1%), followed by intermediate metabolizers (36.4%). Only one patient (< 5%) was identified as being CYP2C9 poor metabolizer (PM). By definition, CYP2C9 PM individuals might be inherently insensitive to DDI in the presence of a (strong) inhibitor of CYP2C9. All subjects but one had an increase in S-warfarin AUC_0-144 h_ on Day 28 (warfarin + ceritinib) relative to Day 1 (warfarin alone) regardless of CYP2C9 phenotype; a similar trend was less apparent for the metabolite 7-hydroxy-S-warfarin (Figure S3). The presence of only 1 PM in the study population precludes any definitive conclusion about the impact of CYP2C9 genetic polymorphisms, particularly of the less prevalent PM phenotype, on the magnitude of DDI between ceritinib and concomitant medications that are substrates of CYP2C9.

**Table 3:** Patient disposition by CYP2C9 metabolizer subtype

Phenotype	Examples of genotypes	Clinical implication^a^	Frequency, *n* (%) (*N* = 33)
Extensive metabolizer	CYP2C9*1/*1	Normal enzyme activity	17 (51.5)
Intermediate metabolizer	CYP2C9*1/*3, *1/*2	Intermediate enzyme activity, highly variable among individuals	12 (36.4)
Poor metabolizer	CYP2C9*2/*2, *3/*3, *2/*3	Low or deficient enzyme activity	1 (3.0)
Unknown^b^	–	–	3 (9.1)

^a^Based on guideline reported by Caudle et al. [17]

^b^Unknown status due to blood specimen not collected and/or genotype testing not performed

### Pharmacokinetics of ceritinib (perpetrator drug)

The average pre-dose (trough) concentration–time profile for ceritinib is shown in Figure S2. On the basis of trough concentrations in plasma after repeated daily dosing, steady-state drug exposure was reached on Day 28 following 3 weeks of dosing and remained stable afterwards (geometric mean *C*_trough_ ranging between 767 and 1040 ng/mL). The results are in line with the known pharmacokinetic properties of ceritinib in crizotinib-naïve and crizotinib pre-treated *ALK* + NSCLC patients at the 750 mg/day fasted dosing regimen [13].

### Summary of pharmacokinetic inhibition of CYP3A- and CYP2C9-mediated metabolism by ceritinib

**Fig. 3:**
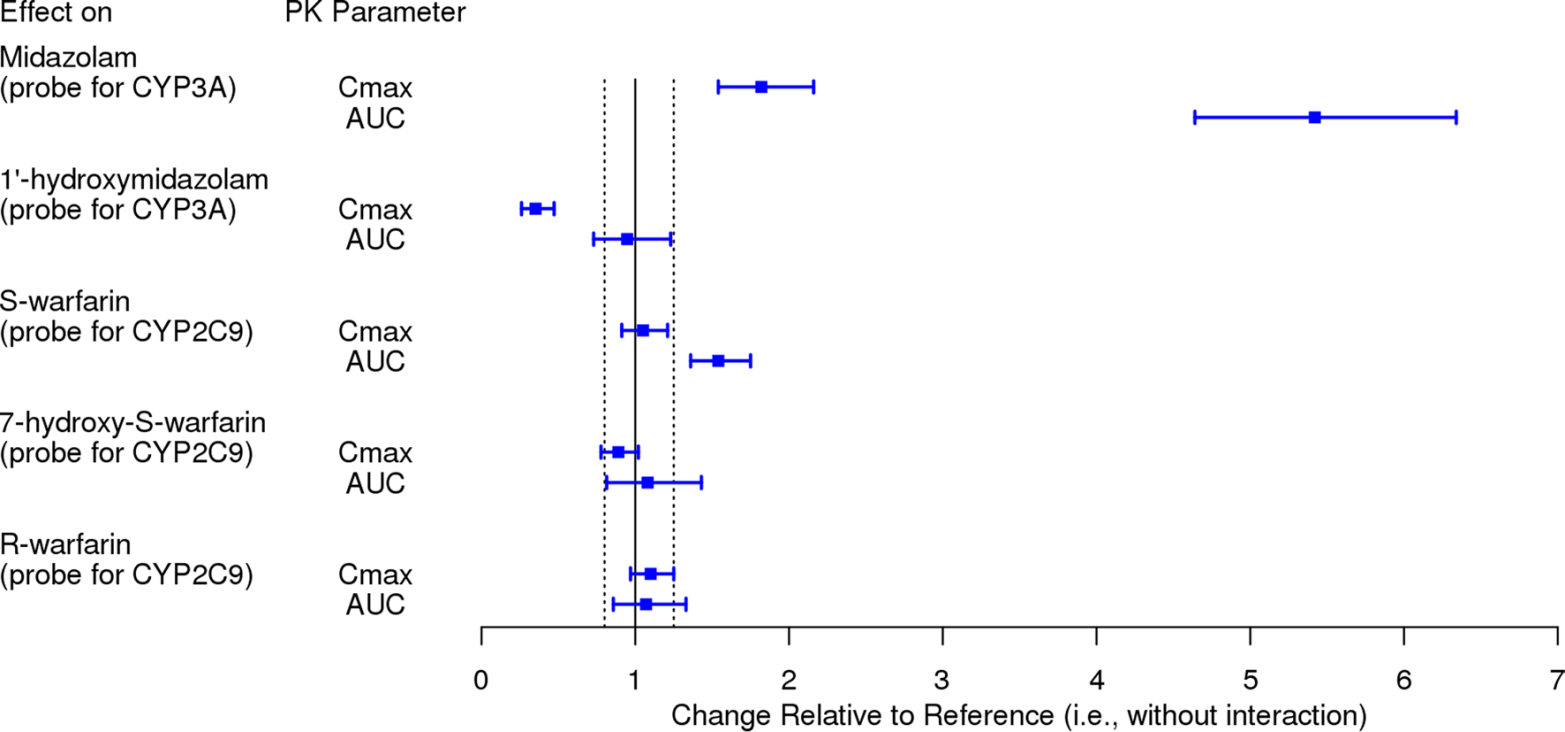
Forest plot illustrating the inhibitory effect of ceritinib on the metabolism of midazolam and warfarin (geometric mean ratio and 90% confidence interval). Dashed vertical lines denote the 0.8 to 1.25 reference interval used to inform dosing recommendations. Refer to Table S2 for a tabular summary of the results. Dosing regimen: midazolam administered as a 2.5 mg single dose; warfarin administered as a 10 mg single dose; ceritinib administered orally at 750 mg QD for 3 weeks. *AUC* area under the plasma concentration–time curve, *C*_max_ maximum plasma concentration

A forest plot illustrating the effect of ceritinib on the pharmacokinetics of selected CYP3A and CYP2C9 probe substrates is provided in Fig. 3. Changes in systemic exposure of midazolam and S-warfarin were primarily used to inform dosing recommendations in the local prescribing information for ceritinib, including USPI and EU SmPC.

## Safety and efficacy of ceritinib

### Safety

The median duration of treatment exposure was 10.14 weeks (range: 0.4–82.4). AEs were reported in 32 (97%) patients; of these, 29 (87.9%) patients had AEs suspected to be study drug related. The most frequently reported AEs regardless of study drug relationship for all patients (≥ 10 patients for all grades) were diarrhea (75.8%), nausea (60.6%), vomiting (51.5%), increased ALT (36.4%), increased GGT (33.3%), increased AST (30.3%), increased ALP (30.3%), and asthenia (30.3%). Grade 3 or 4 AEs suspected to be study drug related were reported in 16 patients (48.5%); the most common (≥ 10%) suspected grade 3/4 AEs were increases in ALT (24.2%), GGT (18.2%), and AST (12.1%) (Table S3). Six patients (18.2%) discontinued treatment due to AEs; the most common AEs leading to study discontinuation were diarrhea (12.1%), nausea (9.1%), vomiting (6.1%), and fatigue (6.1%).

In total, 8 patients died during the study; of these, 4 deaths occurred during the on-treatment period (within 30 days of last dose of ceritinib). None of the deaths were suspected to be treatment related. The cause of all 8 deaths was study disease. Serious AEs were reported in 13 (39.4%) patients, with 11 patients (33.3%) reporting grade 3/4 SAEs. Only 3 patients (9.1%) had SAEs suspected to be related to study drug.

### Efficacy

Overall, ORR was 27.3% (95% CI: 13.3, 45.5) and DCR was 45.5% (95% CI: 28.1, 63.6). Among the 19 patients with NSCLC, ORR was 42.1% (95% CI: 20.3, 66.5), and DCR was 63.2% (95% CI: 38.4, 83.7) (Table S4). The median DOR was not evaluable.

## Discussion

On the basis of early in vitro evidence suggesting that ceritinib may inhibit CYP3A and 2C9 at clinical concentrations, the initial recommendation was to avoid concurrent use of CYP3A and 2C9 substrates known to have narrow therapeutic indices or substrates primarily metabolized by CYP3A and 2C9 during treatment with ceritinib, unless dose reduction of the substrate(s) was considered. Furthermore, experimental IC_50_ and Ki values for ceritinib inhibition of CYP3A (midazolam as substrate) and 2C9 activity (diclofenac as substrate) in HLM relative to the *C*_max_ of ceritinib at steady-state, observed in the first-in-human study, provided early evidence of a clinical drug interaction potential. In light of the potential of ceritinib to increase the systemic exposure of concomitant CYP3A and 2C9 substrates at the recommended dose, a confirmatory clinical DDI study was conducted in patients with cancer to allow for a more definitive determination on dosing concomitant CYP3A and 2C9 substrates with ceritinib. This phase I, multi-center, open-label, single sequence, crossover study evaluated the effect of repeat daily doses of ceritinib on the pharmacokinetics of the probe substrates midazolam and warfarin administered as a two-drug cocktail, which are metabolized by CYP3A and 2C9, respectively. A total of 33 adult patients with advanced cancers harboring genetic alterations in *ALK* including NSCLC participated in the study.

In vitro studies utilizing HLM revealed that ceritinib reversibly inhibits CYP3A4/5 (IC_50_ = 0.2 µM, Ki = 0.16 µM) and CYP2C9 (IC_50_ = 2 µM, Ki = 0.24 µM), among other CYPs of more minor clinical relevance including CYP2A6, 2B6, and 2D6 [8]. Considering a *C*_max_ at steady-state of 1010 ng/mL (or 1.8 µM) observed in earlier clinical trials, the potential for ceritinib to inhibit the metabolism of co-administered CYP3A/2C9 substrates at clinically-relevant doses needed to be further investigated. As ceritinib exhibits nonlinear, time-dependent pharmacokinetics in humans, the latter likely a result of auto-inhibition of CYP3A, [8, 14] ceritinib was dosed repeatedly at 750 mg/day for 3 weeks to promote maximum inhibition of CYP3A/2C9 prior to administration of the midazolam + warfarin cocktail on Day 28, and was continued during the midazolam/warfarin elimination phase (Days 28–34). T

The need for repeat doses of ceritinib in the current DDI study was also supported by physiology-based pharmacokinetic (PBPK) model simulations of the effect of ceritinib on the exposure of midazolam, a sensitive CYP3A substrate. The PBPK model predicted much greater magnitudes of pharmacokinetic interaction when ceritinib was administered to steady-state as compared to single dose ceritinib; when a single dose of ceritinib (750 mg) was co-administered with a single oral dose of midazolam (5 mg), midazolam AUC increased by only 30%, whereas when ceritinib was dosed to steady-state, midazolam AUC following a single oral dose (5 mg) was predicted to increase by 6-fold [8]. Ceritinib is both a substrate and time-dependent inhibitor of human CYP3A in vitro. Because ceritinib features time-dependent clearance in patients, presumably a result of auto-inhibition of CYP3A-mediated metabolism, the smaller change in midazolam exposure with a single ceritinib dose is attributed to incomplete inhibition of CYP3A. Considering that ceritinib achieves steady-state within 2–3 weeks following repeat daily dosing with an accumulation ratio of 6, [8] CYPs are expected to be inhibited to their full extent only after multiple daily doses of ceritinib.

Coadministration of ceritinib increased the AUC of midazolam by 5.42-fold (90% CI: 4.64, 6.34) and *C*_max_ by 1.82-fold (90% CI: 1.54, 2.16) compared to midazolam administered alone, indicating that ceritinib is a strong inhibitor of CYP3A. Furthermore, an 85% decrease in the metabolite-to-parent AUC ratio for 1′-hydroxymidazolam was observed, further confirming a strong inhibitory effect on CYP3A-mediated metabolism of midazolam in the presence of ceritinib. All in all, these results are consistent with the in vitro assessment conducted previously, in which ceritinib showed relatively potent reversible and time-dependent inhibition of CYP34/5 when incubated with midazolam in HLM assay.

Coadministration of CYP3A substrates with strong CYP3A inhibitors, such as ceritinib, often requires inclusion of actionable recommendations in the labeling for the product that is perpetrator of the drug interaction. In humans, CYP3A subfamily enzymes are the most abundant cytochrome enzymes and are predominantly expressed in the liver and intestine, and are involved in the metabolism of a vast number of key therapeutic drugs (e.g., benzodiazepines, calcium channel blockers, protease inhibitors, statins) as well as endogenous compounds (e.g., testosterone, cholesterol). On the basis of new evidence from this clinical pharmacology study, particularly, the major effect of ceritinib on midazolam pharmacokinetics, co-administration of ceritinib with sensitive substrates of CYP3A or CYP3A substrates known to have narrow therapeutic indices (including but not limited to cyclosporine, dihydroergotamine, ergotamine, fentanyl, pimozide, quinidine, tacrolimus, alfentanil, and sirolimus) should be avoided. In cases where concomitant use is unavoidable due to a medical need, dose reduction of the CYP3A substrate(s) with an NTI should be considered by the treating physician, as ceritinib may increase the systemic exposure of such medications to an important extent.

In addition to CYP3A, this study evidenced that ceritinib also inhibits CYP2C9 but to a much lesser extent. CYP2C9 mediates the metabolism of warfarin, phenytoin, diclofenac, ibuprofen, celecoxib, tolbutamide, glimepiride, among other commonly prescribed drugs. In addition, CYP2C9 is subject to clinically important genetic polymorphisms that cause variability in individual drug response including presence of allele genes associated with deficient enzyme activity (CYP2C9*2/*2, *3/*3, and *2/*3, commonly referred to as “poor metabolizers”).

Coadministration of ceritinib increased the AUC_0-inf_ of S-warfarin (the pharmacologically more potent enantiomer) by 54% (90% CI: 36%, 75%) with no change in *C*_max_ compared to warfarin administered alone, indicating that ceritinib is a weak inhibitor of CYP2C9. However, considering the medical relevance of warfarin as a commonly prescribed oral anti-coagulant that has an NTI (i.e., drugs whose exposure–response relationship indicates that minimal concentration changes may lead to serious safety concerns), an exposure increase by 54% is considered clinically important given the potential risk of changing the downstream pharmacodynamic effect of warfarin. Furthermore, an 18% decrease in the metabolite-to-parent AUC ratio for 7-hydroxy-S-warfarin indicates a modest inhibitory effect of ceritinib on CYP2C9-mediated metabolism of warfarin. The impact of ceritinib on R-warfarin pharmacokinetics was negligible (Fig. 2, Figure S1). It is worth noting that the comparative statistical analysis for warfarin pharmacokinetics was performed in the entire dataset that included CYP2C9 extensive, intermediate, and poor metabolizers. As only 1 out of 33 subjects (< 5%) was classified as poor metabolizer (Table 3), inclusion or not of this single subject would have had negligible impact to the overall results.

Overall, the frequency, severity, and type of AEs were consistent with the known safety profile of ceritinib, and no new safety signals were reported [7, 16]. The efficacy results for patients with NSCLC in this heavily pretreated patient population were similar to those observed in previous studies in pretreated patients with *ALK*-positive NSCLC [7, 16].

In summary, we conducted a multi-center, multiple-dose drug interaction study to investigate the inhibitory potential of the ALK-inhibitor ceritinib towards 2 clinically relevant CYP enzymes, 3A4 and 2C9, using a 2-drug cocktail probe approach in 33 cancer patients representative of the target population of ceritinib. The single sequence crossover study design allowed for the robust pharmacokinetic characterization of midazolam, S- and R-warfarin as well as their metabolites to simultaneously assess CYP3A and 2C9 activity following ceritinib co-administration. The results of this study indicate that ceritinib, at the clinically recommended dose, affects the pharmacokinetics of midazolam and warfarin to different extents. On the basis of new pharmacokinetic and safety data, revision of dosing recommendations for concomitant use of ceritinib with CYP3A and 2C9 substrates is warranted. Product labeling should reflect the following:

1. The interaction between ceritinib and CYP3A substrates is of clinical relevance, particularly sensitive substrates (e.g., midazolam, triazolam, alfentanil) or those that have a NTI (e.g., CYP3A substrates known to prolong the QT interval).
2. Concomitant use of ceritinib with sensitive substrates of CYP3A or CYP3A substrates known to have a NTI should be avoided. If concomitant use is unavoidable, dose reduction of the CYP3A substrate(s) should be considered.
3. Concomitant use of ceritinib with sensitive substrates of CYP2C9 or CYP2C9 substrates for which minimal concentration changes may lead to serious toxicities (e.g., warfarin, phenytoin) should be avoided. If concomitant use of such drugs is unavoidable, the prescriber should consider reducing the dose of the coadministered CYP2C9 substrate(s).
4. If coadministration with warfarin is unavoidable due to medical need, it is also recommended to increase the frequency of international normalized ratio (INR) monitoring as the anti-coagulant effect of warfarin may be enhanced while the patient is on treatment with ceritinib.

## Electronic supplementary material

Below is the link to the electronic supplementary material.

Supplementary file1 (PDF 9 kb)

Supplementary file2 (PDF 125 kb)

Supplementary file3 (PDF 120 kb)

Supplementary file4 (PDF 9 kb)

Supplementary file5 (PDF 489 kb)

Supplementary file6 (PDF 383 kb)

Supplementary file7 (PNG 492 kb)

## Data Availability

Novartis is committed to sharing with qualified external researchers, access to patient-level data and supporting clinical documents from eligible studies. These requests are reviewed and approved by an independent review panel on the basis of scientific merit. All data provided are anonymized to respect the privacy of patients who have participated in the trial in line with applicable laws and regulations. This trial data availability is according to the criteria and process described on www.clinicalstudydatarequest.com.
