# Supplementary material for: Effect of ceritinib on the pharmacokinetics of coadministered CYP3A and 2C9 substrates: a phase I, multicenter, drug–drug interaction study in patients with ALK + advanced tumors
Source: Cancer Chemother Pharmacol. 2021 Jan 4;87(4):475–86. doi: 10.1007/s00280-020-04180-3 (PMC7946667; doi:10.1007/s00280-020-04180-3)
Supplement: Supplementary file 1 — Supplementary file1 (PDF 9 kb) [file 280_2020_4180_MOESM1_ESM.pdf]

Table S1. Patient disposition

Disposition/Reason	All Patients<br>N=33<br>n (%)
**DDI phase (pharmacokinetic evaluation)**	
Completed	18 (54.5)
Discontinued from DDI phase	15 (45.5)
Permanently discontinued the study	12 (36.4)
Primary reason for discontinuation from DDI phase	
Adverse event	8 (24.2)
Progressive disease	6 (18.2)
Death	1 (3.0)
**Treatment phase (DDI phase + post-DDI phase)**	
Discontinued from treatment phase	33 (100)
Primary reason for discontinuation from treatment phase	
Adverse event	6 (18.2)
Study terminated by sponsor	12 (36.4)
Progressive disease	13 (39.4)
Subject/guardian decision	1 (3.0)
Death	1 (3.0)
