# Supplementary material for: Effect of ceritinib on the pharmacokinetics of coadministered CYP3A and 2C9 substrates: a phase I, multicenter, drug–drug interaction study in patients with ALK + advanced tumors
Source: Cancer Chemother Pharmacol. 2021 Jan 4;87(4):475–86. doi: 10.1007/s00280-020-04180-3 (PMC7946667; doi:10.1007/s00280-020-04180-3)
Supplement: Supplementary file 2 — Supplementary file2 (PDF 125 kb) [file 280_2020_4180_MOESM2_ESM.pdf]

Table S2. Summary of statistical analysis of primary pharmacokinetic parameters of midazolam and warfarin and their major metabolites, before and after co-administration of ceritinib at 750 mg orally QD for 3 weeks

Pharmacokinetic parameter (unit)	n	Adjusted geometric mean	Treatment comparison: Geometric mean ratio (90% CI)[1]
**CYP3A**			
**Midazolam**			
**$AUC_{0\text{-}inf}$ (ng*hr/mL)**			
midazolam	20	72.5	5.42
midazolam + ceritinib	20	393	(4.64, 6.34)
**C_{max} (ng/mL)**			
midazolam	20	23.0	1.82
midazolam + ceritinib	20	42.0	(1.54, 2.16)
**1'-hydroxymidazolam**			
**$AUC_{0\text{-}inf}$ (ng*hr/mL)**			
midazolam	19	16.2	0.947
midazolam + ceritinib	13	15.4	(0.729, 1.23)
**C_{max} (ng/mL)**			
midazolam	19	5.43	0.351
midazolam + ceritinib	19	1.90	(0.260, 0.472)
**CYP2C9**			
**S-warfarin**			
**$AUC_{0\text{-}inf}$ (ng*hr/mL)**			
warfarin	18	21200	1.54
warfarin + ceritinib	10	32800	(1.36, 1.75)
**C_{max} (ng/mL)**			
warfarin	20	569	1.05
warfarin + ceritinib	19	598	(0.912, 1.21)
**R-warfarin**			
**$AUC_{0\text{-}inf}$ (ng*hr/mL)**			
warfarin	10	36300	1.07
warfarin + ceritinib	4	38900	(0.857, 1.33)
**C_{max} (ng/mL)**			
warfarin	20	560	1.10
warfarin + ceritinib	20	616	(0.969, 1.25)
**7-hydroxy-S-warfarin**			
**$AUC_{0\text{-}inf}$ (ng*hr/mL)**			
warfarin	12	3090	1.08
warfarin + ceritinib	5	3330	(0.814, 1.43)
**C_{max} (ng/mL)**			
warfarin	16	39.1	0.890
warfarin + ceritinib	13	34.8	(0.777, 1.02)

Model was a linear mixed effects model of the log-transformed $AUC_{0\text{-}inf}$ and C_{max}, and included treatment as a fixed effect and patient as a random effect.
Adjusted geometric mean, geometric mean ratio, and 90% confidence interval (90% CI) of $AUC_{0\text{-}inf}$ and C_{max} were all determined from the model and values were back-transformed from log-scale.
$AUC_{0\text{-}inf}$: area under the plasma concentration-time curve t=0-∞; C_{max}: maximum plasma concentration observed; *n*: number of observations included in the analysis.
[1] Probe substrate + ceritinib / probe substrate alone.
