# Supplementary material for: Effect of ceritinib on the pharmacokinetics of coadministered CYP3A and 2C9 substrates: a phase I, multicenter, drug–drug interaction study in patients with ALK + advanced tumors
Source: Cancer Chemother Pharmacol. 2021 Jan 4;87(4):475–86. doi: 10.1007/s00280-020-04180-3 (PMC7946667; doi:10.1007/s00280-020-04180-3)
Supplement: Supplementary file 3 — Supplementary file3 (PDF 120 kb) [file 280_2020_4180_MOESM3_ESM.pdf]

Table S3. All-causality adverse events in ≥10% of patients

Preferred Term	All Patients N=33	
	All Grades n (%)	Grade 3/4 n (%)
Total	32 (97.0)	25 (75.8)
Diarrhoea	25 (75.8)	1 (3.0)
Nausea	20 (60.6)	2 (6.1)
Vomiting	17 (51.5)	3 (9.1)
Increased ALT	12 (36.4)	9 (27.3)
Increased GGT	11 (33.3)	6 (18.2)
Increased AST	10 (30.3)	4 (12.1)
Asthenia	10 (30.3)	2 (6.1)
Increased ALP	10 (30.3)	2 (6.1)
Anaemia	8 (24.2)	2 (6.1)
Decreased appetite	8 (24.2)	1 (3.0)
Abdominal pain	7 (21.2)	1 (3.0)
Blood creatinine increased	7 (21.2)	1 (3.0)
Constipation	7 (21.2)	0
Cough	7 (21.2)	0
Dyspepsia	7 (21.2)	0
Fatigue	7 (21.2)	2 (6.1)
Hypokalaemia	7 (21.2)	3 (9.1)
Weight decreased	7 (21.2)	0
Abdominal pain upper	6 (18.2)	0
Dyspnoea	6 (18.2)	2 (6.1)
Headache	6 (18.2)	0
Non-cardiac chest pain	6 (18.2)	0
Rash	6 (18.2)	0
Electrocardiogram QT prolonged	5 (15.2)	0
Dizziness	4 (12.1)	0
Hypertension	4 (12.1)	0
Lipase increased	4 (12.1)	3 (9.1)
Pyrexia	4 (12.1)	0

ALP: alkaline phosphatase; ALT: alanine aminotransferase; AST: aspartate aminotransferase; GGT: gamma-glutamyl transferase.
