# Supplementary material for: Effect of ceritinib on the pharmacokinetics of coadministered CYP3A and 2C9 substrates: a phase I, multicenter, drug–drug interaction study in patients with ALK + advanced tumors
Source: Cancer Chemother Pharmacol. 2021 Jan 4;87(4):475–86. doi: 10.1007/s00280-020-04180-3 (PMC7946667; doi:10.1007/s00280-020-04180-3)
Supplement: Supplementary file 4 — Supplementary file4 (PDF 9 kb) [file 280_2020_4180_MOESM4_ESM.pdf]

**Table S4.** Best overall response per investigator assessment (per RECIST 1.1)

| Best Overall Response | NSCLC N=19 | Other N=14 | All Patients N=33 |
|---|---|---|---|
| Complete response (CR), n (%) | 1 (5.3) | 0 | 1 (3.0) |
| Partial response (PR), n (%) | 7 (36.8) | 1 (7.1) | 8 (24.2) |
| Stable disease (SD), n (%) | 2 (10.5) | 2 (14.3) | 4 (12.1) |
| Progressive disease (PD), n (%) | 3 (15.8) | 9 (64.3) | 12 (36.4) |
| Non-CR/Non-PD, n (%) | 2 (10.5) | 0 | 2 (6.1) |
| Unknown, n (%) | 4 (21.1) | 2 (14.3) | 6 (18.2) |
| Overall response rate (ORR: CR + PR), n (%) [95% CI] | 8 (42.1) [20.3, 66.5] | 1 (7.1) [0.2, 33.9] | 9 (27.3) [13.3, 45.5] |
| Disease control rate (DCR: CR + PR + SD + non-CR/non-PD) | 12 (63.2) [38.4, 83.7] | 3 (21.4) [4.7, 50.8] | 15 (45.5) [28.1, 63.6] |
