# Supplementary figures and images for: Effect of ceritinib on the pharmacokinetics of coadministered CYP3A and 2C9 substrates: a phase I, multicenter, drug–drug interaction study in patients with ALK + advanced tumors
Source: Cancer Chemother Pharmacol. 2021 Jan 4;87(4):475–86. doi: 10.1007/s00280-020-04180-3 (PMC7946667; doi:10.1007/s00280-020-04180-3)

**A**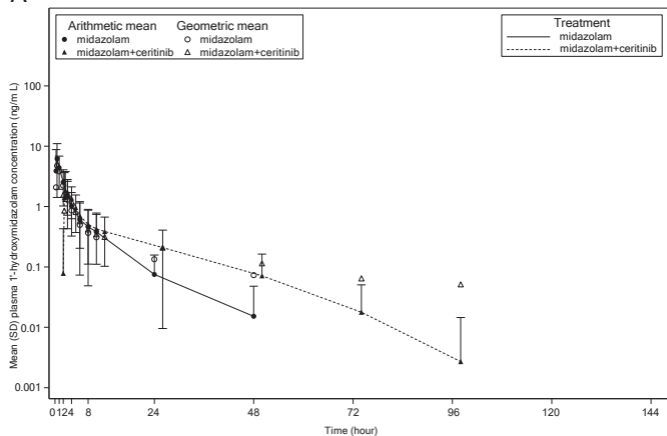**B**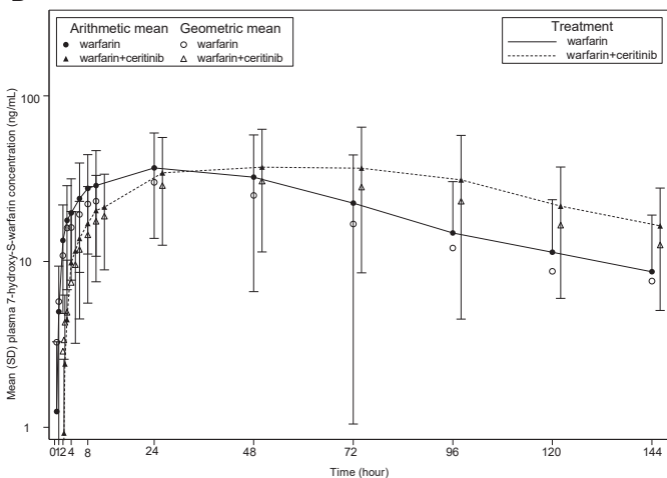**C**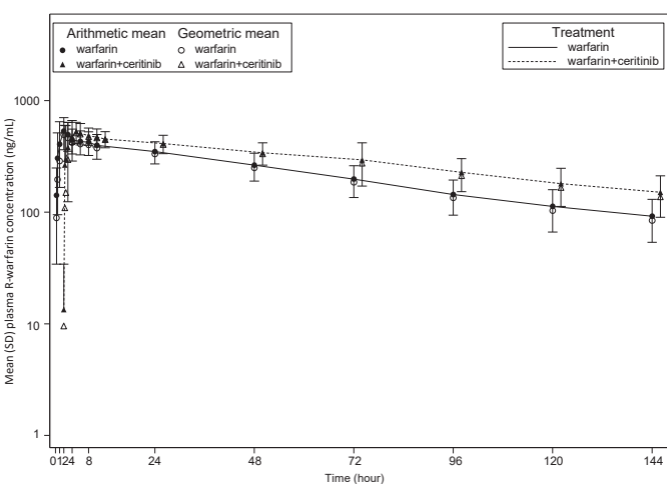

Supplement: Supplementary file 5 — Supplementary file5 (PDF 489 kb) [file 280_2020_4180_MOESM5_ESM.pdf]

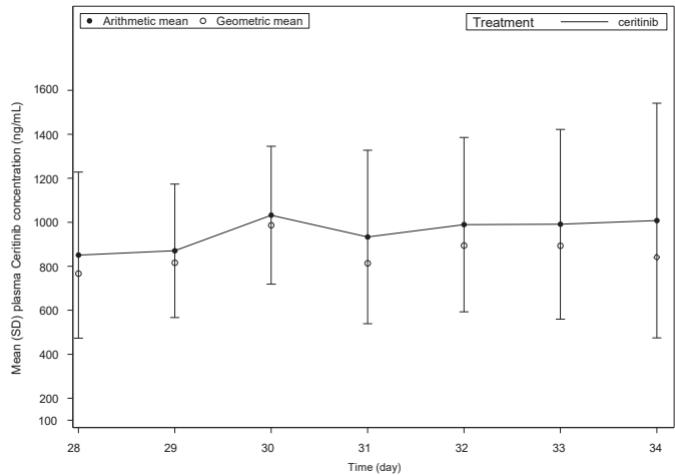

Supplement: Supplementary file 6 — Supplementary file6 (PDF 383 kb) [file 280_2020_4180_MOESM6_ESM.pdf]

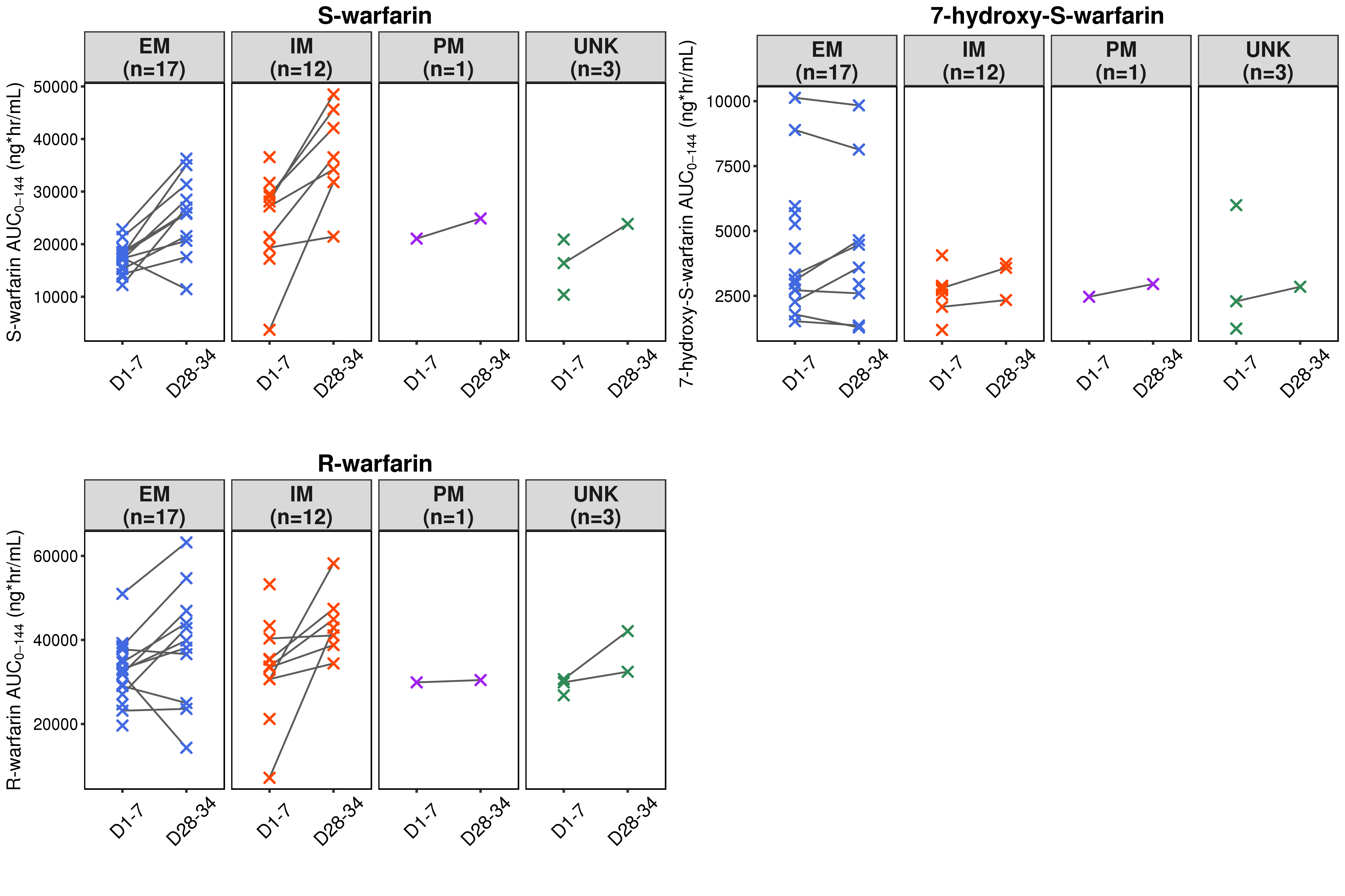

Supplement: Supplementary file 7 — Supplementary file7 (PNG 492 kb) [file 280_2020_4180_MOESM7_ESM.png]
